# Oxysterol 7-α Hydroxylase (CYP7B1) Attenuates Metabolic-Associated Fatty Liver Disease in Mice at Thermoneutrality

**DOI:** 10.3390/cells10102656

**Published:** 2021-10-05

**Authors:** Ioannis Evangelakos, Dorothee Schwinge, Anna Worthmann, Clara John, Niklas Roeder, Paul Pertzborn, Janina Behrens, Christoph Schramm, Ludger Scheja, Joerg Heeren

**Affiliations:** 1 Department of Biochemistry and Molecular Cell Biology, University Medical Center Hamburg-Eppendorf, 20246 Hamburg, Germany; i.evangelakos@uke.de (I.E.); a.worthmann@uke.de (A.W.); clara_john@gmx.de (C.J.); nproeder@googlemail.com (N.R.); paulpertzborn@gmx.de (P.P.); janina.behrens.jb@googlemail.com (J.B.); l.scheja@uke.de (L.S.); 2 Department of Medicine, University Medical Center Hamburg-Eppendorf, 20246 Hamburg, Germany; d.schwinge@uke.de (D.S.); c.schramm@uke.de (C.S.)

**Keywords:** bile acids, hydroxycholesterol, oxysterol, Cyp7b1, non-alcoholic fatty liver disease, T cells, inflammation, fibrosis, steatohepatitis, metabolic-associated fatty liver disease

## Abstract

Ambient temperature is an important determinant of both the alternative bile acid synthesis pathway controlled by oxysterol 7-α hydroxylase (CYP7B1) and the progression of metabolic-associated fatty liver disease (MAFLD). Here, we investigated whether CYP7B1 is involved in the etiology of MAFLD under conditions of low and high energy expenditure. For this, Cyp7b1^−/−^ and wild type (WT) mice were fed a choline-deficient high-fat diet and housed either at 30 °C (thermoneutrality) or at 22 °C (mild cold). To study disease phenotype and underlying mechanisms, plasma and organ samples were analyzed to determine metabolic parameters, immune cell infiltration by immunohistology and flow cytometry, lipid species including hydroxycholesterols, bile acids and structural lipids. In WT and Cyp7b1^−/−^ mice, thermoneutral housing promoted MAFLD, an effect that was more pronounced in CYP7B1-deficient mice. In these mice, we found higher plasma alanine aminotransferase activity, hyperlipidemia, hepatic accumulation of potentially harmful lipid species, aggravated liver fibrosis, increased inflammation and immune cell infiltration. Bile acids and hydroxycholesterols did not correlate with aggravated MAFLD in Cyp7b1^−/−^ mice housed at thermoneutrality. Notably, an up-regulation of lipoprotein receptors was detected at 22 °C but not at 30 °C in livers of Cyp7b1^−/−^ mice, suggesting that accelerated metabolism of lipoproteins carrying lipotoxic molecules counteracts MAFLD progression.

## 1. Introduction

Metabolic-associated fatty liver disease (MAFLD) [1] is a disease with a high burden and steadily increasing prevalence in the Western world (~25% of global population) [2], which is closely associated with obesity, insulin resistance, dyslipidemia and type 2 diabetes [3]. Starting from simple hepatic steatosis, the disease can progress to severe steatohepatitis (known also as non-alcoholic steatohepatitis (NASH)) that is characterized by additional hepatic injury, inflammation and fibrosis [4]. Despite major advances in understanding the pathogenesis of MAFLD, the molecular mechanisms that drive the transition towards chronic inflammation and fibrosis are still incompletely understood. When it comes to prevention strategies against MAFLD and associated diseases, a great emphasis is given on the reduction of circulating as well as hepatic lipids including cholesterol [5]. Cholesterol accumulation or dysregulation of its metabolism has been correlated with MAFLD in humans as well as in murine models of the disease [6]. Next to the classical bile acid synthesis pathway that is controlled by a negative feedback regulating the key enzyme cholesterol 7-α hydroxylase (CYP7A1), cholesterol can also be converted to bile acids via specific hydroxycholesterol intermediates in the alternative pathway [7]. These cholesterol derivatives are 25-hydroxycholesterol (25-HC) (generated in peripheral organs by the enzyme cholesterol 25-hydroxylase) and 27-HC (primarily generated in the liver by mitochondrial sterol 27-hydroxylase (CYP27A1)). The subsequent 7-α hydroxylation of 25-HC and 27-HC by oxysterol 7-α hydroxylase (CYP7B1) is then the defining step of the alternative bile acid synthesis pathway [7]. Recently, we demonstrated that housing mice at a low environmental temperature is characterized by higher flux of cholesterol to the liver [8,9], where the induction of CYP7B1 facilitates the conversion to bile acids [10]. This previously unappreciated homeostatic mechanism protects from peripheral and hepatic cholesterol accumulation and could be beneficial against fatty liver and MAFLD [11].

Oxysterols and bile acids have been described to modulate immune cell plasticity and differentiation. For instance, 25-HC has been shown to inhibit the type 1 interferon response via a negative feedback [12], while 27-HC stimulates pro-inflammatory processes via interaction with the estrogen receptor [13]. Moreover, the binding of oxidized cholesterol derivatives to the retinoic acid receptor-related orphan receptor γ (RORγt) stimulates the differentiation of TH17 cells [14]. Further modification to sulfated sterol derivatives resulted in enhanced RORγt binding while Th17 inhibitory sterol receptor liver X receptor (LXR) activity was reduced by these metabolites [15,16]. Recent papers indicate that specific bile acid species also modulate immune cell function. For example, the secondary bile acid 3-oxo lithocholic acid (LCA) suppressed the differentiation of regulatory Th17 cells, while isoallo-LCA promoted the differentiation of regulatory T cells via activation of the master transcriptional regulator Foxp3 [17]. Moreover, it was reported that diet-dependent induction of a distinct population of Foxp3-positive Treg cells is regulated by secondary bile acids via activation of the vitamin D receptor [18]. Next to nuclear receptors, G protein-coupled bile acid receptors (such as TGR5) regulate immune cells in various organs. For instance, the TGR5 ligand LCA reduces pro-inflammatory signaling pathways in macrophages of the liver [19]. Notably, CYP7B1 influences concentrations of hydroxycholesterol and bile acid species, suggesting that the enzyme could affect immune cell abundance and plasticity in chronic inflammatory metabolic disorders such as NASH.

In this context, it has been increasingly recognized that conventional housing temperature (22 °C) exposes mice to a mild cold stress that requires substantial energy expenditure to maintain body core temperature, favors metabolic homeostasis and protects against the development of metabolic disorders [20,21]. In contrast, an ambient temperature of approximately 30 °C is thermoneutral for mice, meaning that no extra energy is required for heat production. This condition has recently been recognized to promote metabolic disease development in mice, which allows better translation of experimental observations to humans who usually prefer to stay warm [20,22]. Supporting the above, it was recently shown that mild cold stress (22 °C) halts the progression of MAFLD whereas thermoneutrality (30 °C) exacerbates its development and leads to a more human-like liver phenotype [23]. These studies highlight the relevance of housing temperatures when studying metabolic disease in mice, as high energy expenditure observed at regular housing temperature could mask disease mechanisms that are present in humans.

The aim of this study was to investigate how substrates and metabolites of CYP7B1 can affect diet-induced MAFLD under conventional as well as thermoneutral conditions. Here, we show that steatohepatitis and fibrosis was more pronounced at 30 °C. At conventional room temperature, Cyp7b1^−/−^ and wild type littermate controls have a similar phenotype. In contrast, at thermoneutrality MAFLD progression is more severe in Cyp7b1^−/−^ mice than in controls. This phenotype correlates with markers of insulin resistance, distinct changes in the hydroxycholesterol pattern and liver infiltration of pro-inflammatory immune cells. Overall, these results indicate that CYP7B1 protects against diet-induced MAFLD progression in mice housed at thermoneutrality.

## 2. Materials and Methods

### 2.1. Experimental Animals, Housing Conditions, Diets and Animal Experiments

All mouse experiments were approved by the Animal Welfare Officers of University Medical Centre Hamburg-Eppendorf (UKE) and Behörde für Gesundheit und Verbraucherschutz Hamburg (G96/15). Cyp7b1^−/−^ mice were bred and housed in the animal facility of the UKE at 22 °C under a day-night cycle of 12 h and ad libitum access to drinking water and regular chow diet (P1324, Altromin, Germany). For our study, male age-matched Cyp7b1^−/−^ and WT littermates (11–12 weeks-old in the beginning of the experiment) were fed for a period of 8 months with a choline deficient high fat diet (CD-HFD, Research Diets; D05010402). At the end of the study, EDTA blood was harvested after a 4 h fasting period. Subsequently, animals were perfused with PBS and tissues were either conserved in TriFast reagent (Peqlab, Erlangen, Germany) for RNA analysis, fixed in 3.7% formaldehyde solution for histology or snap frozen and stored at −80 °C for lipid and protein analysis. Body composition analysis was performed 1 week before the end of the experiment by echoMRI.

### 2.2. Plasma Parameters

Plasma was isolated by centrifugation of EDTA-spiked blood for 10 min at 10.000 rpm at 4 °C in a bench top centrifuge. Plasma cholesterol and triglycerides were determined using commercial colorimetric kits (Roche, Manheim, Germany) that were adapted to 96-well microtiter plates with Precipath (Roche, Manheim, Germany) as standard. Free fatty acids (FFAs) were determined using the NEFA-HR(2) Assay (FUJIFILM). For lipoprotein profiling, 200 µL plasma was separated by fast-performance liquid chromatography (FPLC) on a Superose 6 10/300 GL column (GE Healthcare, München, Germany) with a flow rate of 0.5 mL/min. Fractions were collected and triglyceride and cholesterol concentrations were measured as described above. Plasma insulin levels were measured with an ELISA kit (#90060, ChrystalChem, Zaandam, The Netherlands) according to the manufacturers protocol. For assessment of liver damage, plasma activity of alanine aminotransferase (ALT) was determined using a photometric ALT Activity Assay (MAK052, Sigma-Aldrich, Darmstadt, Germany). Plasma albumin levels were quantified photometrically with reagents provided by Siemens Healthcare [24].

### 2.3. Bile Acid Measurement

Bile acids were quantified by HPLC coupled to electrospray ionization tandem mass spectrometry [25]. Briefly, portal plasma samples were prepared by a simple methanol liquid-liquid extraction. Quantitative measurement of bile acids was performed using a LC-ESI-QqQ system run multiple reaction monitoring (MRM) mode. HPLC analysis was performed using NEXERA X2 LC-30AD HPLC PUMP (Shimadzu, Tokyo, Japan) equipped with a Kinetex C18 column (100 Å, 150 mm × 2.1 mm i.d., Phenomenex, Torrance, CA, USA). For HPLC a mobile phase A consisting of water and a mobile phase B consisting of acetonitrile methanol (3/1 *v/v*) both enriched with 0.1% formic acid and 20 mM ammonium acetate was used. The column was coupled to QqQ: Q trap 5500 System (SCIEX, Darmstadt, Germany). Peaks were identified and quantified by comparing retention times, as well as MRM transitions and peak areas, respectively, to particular corresponding standard chromatograms.

### 2.4. Liver Lipid and Hydroxycholesterol Analysis

Lipid analysis of structural and storage lipids was performed using the Lipidyzer platform from SCIEX (Framingham, MA, USA). Briefly, snap-frozen liver samples were homogenized and extracted using MTBE/methanol. Lipidyzer Internal Standards were added to all samples during lipid extraction. Lipid extracts were concentrated and reconstituted in running buffer containing 10 mM ammonium acetate, dichloromethane/methanol (50/50). Lipid species were separated by SELEX ion technology and quantified using an ESI-QqQ system operated in multiple reaction monitoring (MRM) mode (QTRAP 5500; SCIEX) employing the Lipidyzer software. Hepatic hydroxycholesterols were analyzed after derivatization with Girard *p* reagent as previously described [26]. Targeted analysis via HPLC coupled to electrospray ionization tandem mass spectrometry was subsequently performed. Briefly, liver samples (approx. 100–150 mg) were homogenized in chloroform/methanol (1:1, *v/v*) and the resulting extracts were vacuum-dried. After resuspension in ethanol, samples were fractionated using reversed phase solid phase extraction (SPE) to enrich oxysterols devoid of cholesterol (Sep-Pak cartridges, Waters, Milford, MA, USA). Subsequently, oxysterols were derivatized with Girard P reagent followed by another SPE clean-up to deplete excess Girard P reagent. Oxysterols were separated on a hypersil GOLD reversed phase column (50 × 2.1 mm, 1.9 µm; Thermo Fisher Scientific, Waltham, MA, USA) using 49.9% H_2_O, 33.3% methanol, 16.7% acetonitrile and 0.1% formic acid as mobile phase A and 4.9% H_2_O, 63.3% methanol, 31.7% acetonitrile and formic acid 0.1% as mobile phase B. The HPLC (NEXERA X2 LC-30AD HPLC PUMP; Shimadzu, Tokyo, Japan) was coupled to a QqQ: Q trap 5500 System (SCIEX, Darmstadt, Germany), run in MRM mode. Quantification was achieved by comparing retention times, MRM transitions and peak areas to corresponding standard chromatograms.

### 2.5. Gene Expression Analysis

Total RNA was isolated using a NucleoSpin RNA II kit (Macherey & Nagel, Düren Germany). After synthesis of cDNA with SuperScript III Reverse Transcriptase (Invitrogen, Waltham, MA, USA), quantitative real-time PCR was performed and the relative expression of genes of interest was calculated by normalization to housekeeper TATA-box binding protein (Tbp). The following TaqMan Assay-on-Demand primer sets (Applied Biosystems, Waltham, MA, USA) were used: Tbp: Mm00446973_m1; Cyp7a1: Mm00484150_m1; Cyp7b1: Mm00484157_m1; Cyp27a1: Mm00470430_m1; Cyp8b1: Mm00501637_s1; Baat: Mm00476075_m1; Abcb11: Mm00445168_m1; Nr0b2 (Shp): Mm00442278_m1; Ch25h: Mm00515486_s1; Il1b: Mm00434228_m1; Il6: Mm00446190_m1; Cxcl1: Mm00433859_m1; Cxcl9: Mm00434946_m1; Cxcl10: Mm00445235_m1; Tnf: Mm00443258_m1; Ccl2: Mm00441242_m1; Ccl5: Mm01302428_m1; Cd68: Mm03047343_m1; Acta2: Mm00725412_s1; Tgfb1: Mm00441724_m1; Col1a1: Mm00801666_g1; Timp1: Mm00441818_m1; Mmp13: Mm00439491_m1; Mmp12: Mm00500554_m1; Fasn: Mm00662319_m1; Scd1: Mm00772290_m1; Acaca: Mm01304285_m1; Pparg: Mm00440945_m1: Abcg5: Mm00446249_m1; Abcg8: Mm00445970_m1; Ldlr: Mm00440169_m1; Lrp1: Mm00464608_m1; Sult2a1: Mm04205657_mH; Sult2b1: Mm00450550_m1; Trem2: Mm00451744_m1. Cd9 and Gpnmb expression were assessed using SYBR green primers which were premixed with SYBR Green PCR Master Mix (Applied Biosystems). The following primer pairs were used: Cd9 forward primer: 5′- TGCTGGGATTGTTCTTCGGG-3′, Cd9 reverse primer: 5′- GCTTTGAGTGTTTCCCGCTG-3′, Gpnmb forward primer: 5′-GAGCACAACCAATTACGTGGCT-3′, Gpnmb reverse primer: 5′-GGTGATATTGGAACCCACCAGA-3′, Tbp forward primer: 5′-CTTCCTGCCACAATGTCACAG-3′, Tbp reverse primer: 5′-CCTTTCTCATGCTTGCTTCTCTG-3′.

### 2.6. Western Blot Analysis

For SDS–PAGE samples were homogenized in a tissue lyser (Qiagen) by adding a 10× fold excess of (*v/w*) RIPA buffer (50 mM Tris-HCl pH 7.4, 5 mM EDTA, 150 mM sodium chloride, 1 mM sodium pyrophosphate, 1 mM sodium fluoride, 1 mM sodium orthovanadate, 1% NP-40) supplemented with cOmplete™ Mini Protease Inhibitor Cocktail (Roche) and Phosphatase Inhibitor Cocktail (Bimake.com). Lysates were centrifuged at 16,000× *g* for 10 min, the clear soluble middle layer without the fat was taken, and protein concentration was determined using an adapted Lowry method [8]. 20 µg of total protein in NuPAGE reducing sample buffer (Invitrogen) were denatured at 60 °C for 10 min and separated on 10% SDS-polyacrylamide Tris-glycine gels. Transfer to nitrocellulose membranes was performed in a wet blotting system, membranes were stained with Ponceau S (Serva) to confirm equal loading, washed twice in TBS-T (20 mM Tris, 150 mM sodium chloride, 0.1% (*v/v*) Tween 20) and blocked in 5% milk powder (Sigma) in TBS-T for 1 h at room temperature. Primary antibody incubation (5% BSA in TBS-T) was performed overnight at 4 °C. Secondary antibodies were diluted in 5% milk powder in TBS-T, and detection was performed with enhanced chemiluminescence using an Amersham Imager 600 (GE Healthcare). The following primary antibodies were used: LDLR (rabbit monoclonal antibody; 1:1000; abcam cat. No. ab52818), CYP7B1 (rabbit monoclonal antibody; 1:1000; abcam cat. No. ab138497) and γ-Tubulin (rabbit monoclonal; 1:2000; cat. No. ab179503). The goat-anti-rabbit HRP coupled secondary antibody from Jackson Immunoresearch (#111-035-144) was used in a dilution of 1:5000.

### 2.7. Histology and Immunohistochemistry

For histology, liver tissues were fixed in 3.7% formaldehyde in PBS solution. Hematoxylin/Eosin, Sirius Red as well as immunohistochemistry (IHC) stainings were performed using 4 μm sections of paraffin-embedded tissues generated by standard procedures. For IHC stainings, rat-anti-Ly6c (ab15627, abcam), rat-anti-F4/80 (MCA497, BIORAD) and rat-anti-CD3 (MCA 1477, BIORAD) primary antibodies were used, all in a dilution of 1:200 in 3% BSA (Sigma). Horseradish peroxidase (HRP) coupled donkey-anti-rat (Jackson Immunoresearch, # 712-036-153) was used as secondary antibody. After secondary antibody incubation, sections were washed with PBS for 3× 10 min. Staining was performed using an abcam DAB kit following the manufacturer’s instructions. After DAB-staining, the slides were rinsed with PBS to stop the chromogenic reaction and counterstained with hematoxilin for 2 min. Slides were incubated under running tap water for 10 min to achieve bluing of the hematoxilin. Afterwards, the slides were dehydrated and mounted using Eukitt. Images were taken using a NikonA1 Ti microscope equipped with a DS-Fi-U3 brightfield camera. The Sirius Red- and F4/80-positive areas were quantified in at least six fields per section using Adobe Photoshop and ImageJ in a blinded manner as described [27]. Ly6c positive cells were quantified by counting the number of stained cells in at least six fields per section in a blinded manner.

### 2.8. Immune Cell Isolation and Staining

Spleen immune cells and liver lymphocytes were isolated as described previously [28]. Briefly, spleens were meshed through a 100 µm cell-strainer in order to get a single cell suspension. Erythrocytes were lysed by short-term incubation of spleen cells in ACK-buffer. For the isolation of liver non-parenchymal cells (NPCs), mouse livers were perfused with PBS and mechanically dissected trough a 100 µm cell-strainer. Hepatocytes and debris were sedimented twice at 40× *g* and non-parenchymal cells were recovered by centrifugation over a 35% Optiprep (Sigma-Aldrich, Taufkirchen, Germany) gradient at 400× *g*. Immunofluorescence surface staining was performed with antibodies against CD3, CD4, CD8, NK1.1, CD45.2, CD11b, CD11c, Ly6C and Ly6G (all BioLegend, Koblenz, Germany). Dead cells were stained with Pacific Orange Succinimidyl Ester (Thermo Fisher Scientific, Germany) and excluded from further analysis. For intracellular cytokine staining, cells were restimulated with PMA (10 ng/mL) and ionomycin (1 µg/mL, both Sigma-Aldrich, Germany) in the presence of GolgiPlug (1 µL/mL, Heidelberg, Germany) for 4 h. Cells were PFA fixed and perforated in PBS containing 0.5% saponine and 2% BSA and stained for IL-17, IFNgamma and TNF (all BioLegend, Koblenz, Germany). Flow cytometry was performed using a BD LSR II cytometer (BD Biosciences, Heidelberg, Germany) and data were analyzed with FACS DIVA software.

### 2.9. Statistics

Statistical analysis was performed with Graph Pad Prism 7.0, where two-way ANOVA followed by post hoc testing (Tukey correction) was conducted. No statistical method was used to predetermine sample size. The statistical parameters (i.e., *p* values, numbers of biological repeats) can be found in the figure legends. No exclusion or inclusion criteria were used for data analyses. *p* < 0.05 was considered significant.

## 3. Results

### 3.1. CYP7B1 Deficiency Exacerbates the Metabolic Phenotype at Thermoneutral Housing

**Figure 1:**
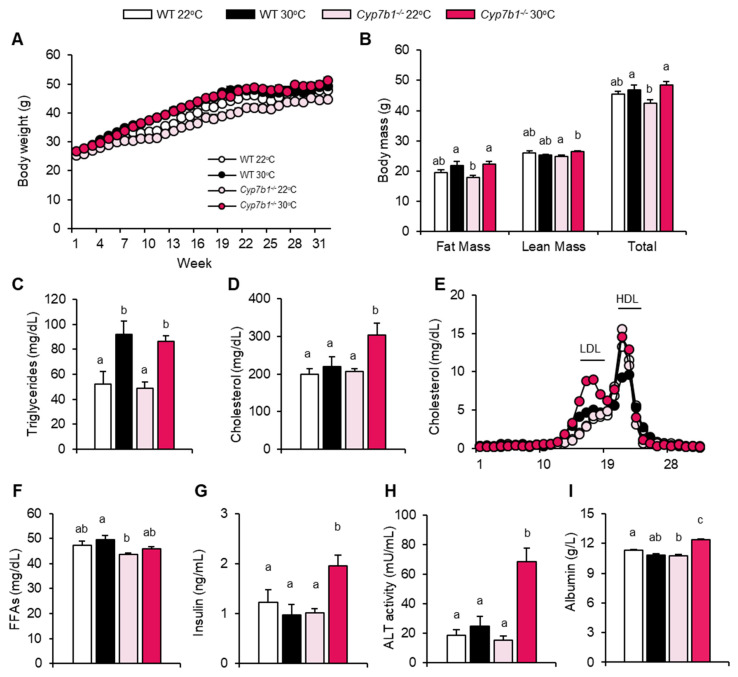
CYP7B1-deficiency induces MAFLD-associated metabolic parameters at thermoneutral housing conditions. Cyp7b1^−/−^ and WT littermates housed under 22 °C or 30 °C were fed a CD-HFD for 8 months. (**A**) Body weights. (**B**) Body composition analysis by echoMRI. (**C**) Triglycerides, (**D**) cholesterol, (**E**) cholesterol in FPLC fractions, (**F**) free fatty acids (FFA), (**G**) insulin, (**H**) alanine aminotransferase (ALT) activity, and (**I**) albumin in plasma collected at the end of the feeding study. Data are shown as mean values ± SEM (number of mice: WT 22 °C: n = 11; WT 30 °C: n = 9; Cyp7b1^−/−^ 22 °C: n = 16; Cyp7b1^−/−^ 30 °C: n = 13). Different letters indicate significant differences between groups (*p* < 0.05) determined by two-way ANOVA.

To investigate if CYP7B1 affects MAFLD progression to steatohepatitis, wild type (WT) and Cyp7b1^−/−^ mice were fed a choline-deficient high fat diet (CD-HFD) commonly used to study diet-induced steatohepatitis [29] for a period of 8 months. To elucidate whether housing temperature modulates the role of CYP7B1 during the disease’s progression, littermates from both groups were housed at room temperature (22 °C) or at thermoneutrality (30 °C). Although all groups started with similar body weights, mice housed at 30 °C initially gained more weight than those at 22 °C (Figure 1A). Compared to mice housed at 22 °C, food intake was lower in mice housed at 30 °C but not different between the genotypes (Figure S1A). After 32 weeks of the CD-HFD intervention, body weight was still different between the two groups of Cyp7b1^−/−^ mice but not the WT mice (Figure 1B). EchoMRI body composition analysis revealed that this was attributed to both fat and lean mass (Figure 1B). Plasma triglycerides were significantly higher in both WT and Cyp7b1^−/−^ mice housed at 30 °C compared to the 22 °C-housed littermates (Figure 1C), an effect likely attributed to reduced lipid clearance by less active brown adipose tissue [30,31]. In line with this notion, thermogenic gene expression analysis of brown adipose tissues showed that Ucp1 and Elovl3 transcript levels were significantly lower in both genotypes upon thermoneutral housing (Figure S1B), while for Dio2 and Ppargc1a this effect of thermoneutrality was only observed in Cyp7b1^−/−^ mice (Figure S1B). Notably, plasma cholesterol levels were significantly higher in thermoneutral housed Cyp7b1^−/−^ but not WT mice (Figure 1D). FLPC analysis showed that this was attributed mainly to the LDL lipoprotein fraction (Figure 1E). Plasma free fatty acids followed a similar, yet not significant, trend, as triglycerides and were slightly higher at thermoneutrality (Figure 1F). Similar to elevated cholesterol and LDL levels, thermoneutral housed Cyp7b1^−/−^ had higher insulin (Figure 1G), plasma alanine aminotransferase activity (Figure 1H) and albumin (Figure 1I) levels. Increased albumin levels may be explained by more pronounced inflammation (see below), a state that has been shown to be associated with a prolonged plasma half-life of albumin through higher expression of the neonatal Fc receptor [32].

Together, plasma parameters indicate that at thermoneutral (but not conventional) housing temperature, CYP7B1 deficiency enhances insulin resistance, dyslipidemia and liver damage in mice fed MAFLD-inducing diet.

### 3.2. Hepatic Lipid Accumulation and Fibrosis Are Increased in CYP7B1-Deficient Mice Housed at Thermoneutrality

**Figure 2:**
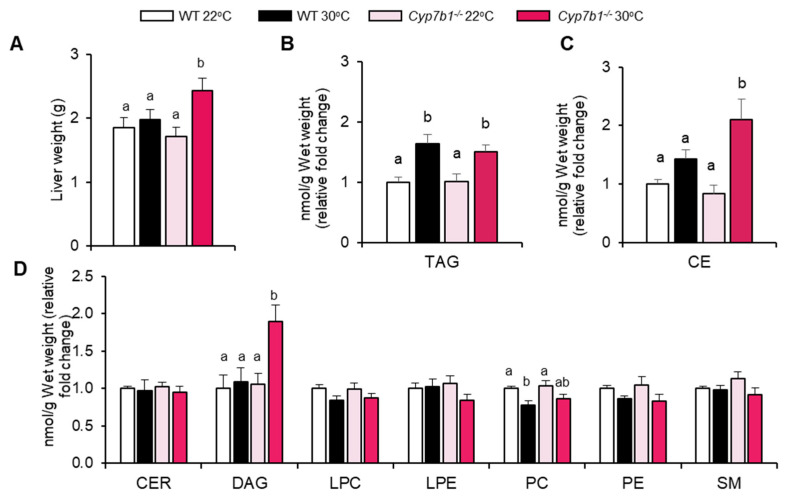
Hepatic accumulation of specific lipid classes in CYP7B1-deficient mice housed at thermoneutrality. Cyp7b1^−/−^ and WT littermates housed under 22 °C or 30 °C were fed a CD-HFD for 8 months. (**A**) Liver weight. (**B**) Hepatic trigylcerides (TAG) and (**C**) cholesteryl esters (CE). (**D**) Hepatic ceramides (CER), diacylglycerols (DAG), lysophosphatidylcholines (LPC), lysophosphatidylethanolamines (LPE), phosphatidylcholines (PC), phosphatidylethanolamines (PE), and sphingomyelins (SM). Data are shown as mean values ± SEM (n = 8), different letters indicate significant differences between groups (*p* < 0.05) determined by two-way ANOVA.

Next, we studied the pathophysiological consequences of housing temperature and CYP7B1 deficiency for the liver. Notably, Cyp7b1^−/−^ mice housed at 30 °C but not 22 °C had higher liver weights than the corresponding WT mice (Figure 2A). Hepatic triglyceride content was higher in mice housed at 30 °C compared to 22 °C, independent of the genotype (Figure 2B). In contrast, cholesteryl esters (Figure 2C) and diacylglycerols but no other lipid classes (Figure 2D) were exclusively higher in livers of Cyp7b1^−/−^ mice at thermoneutrality.

**Figure 3:**
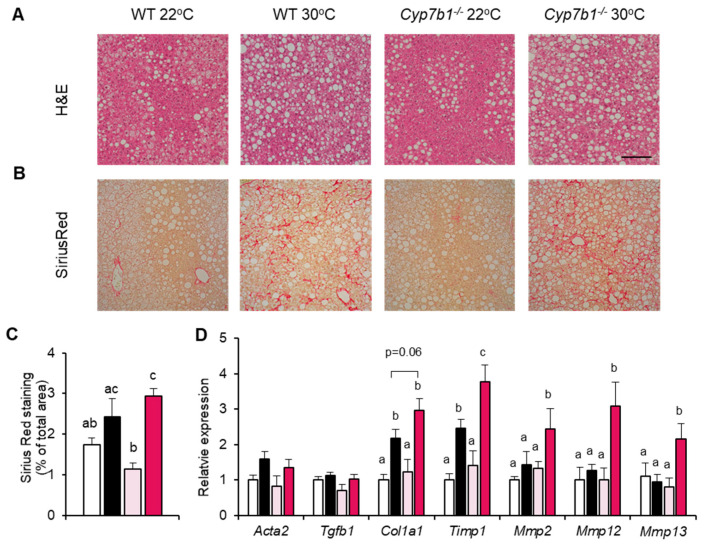
Increased fibrosis in CYP7B1-deficient mice housed at thermoneutrality. Cyp7b1^−/−^ and WT littermates housed under 22 °C or 30 °C were fed a CD-HFD for 8 months. Representative images of (**A**) HE and (**B**) Sirius red stained liver sections. (**C**) Quantification of fibrotic areas (number of mice: WT 22 °C: n = 11; WT 30 °C: n = 9; Cyp7b1^−/−^ 22 °C: n = 16; Cyp7b1^−/−^ 30 °C: n = 13). (**D**) Hepatic expression of fibrosis-related genes normalized to Tbp as a housekeeper (n = 8). Data are shown as mean values ± SEM. Different letters indicate significant differences between groups (*p* < 0.05) determined by two-way ANOVA.

Consistent with the hepatic triglyceride levels (Figure 2B), Cyp7b1^−/−^ and WT mice housed at 30 °C exhibited a higher content of liver lipid droplets, as assessed by histology (Figure 3A). Hepatic collagen deposition, as evaluated by Sirius Red staining (Figure 3B), was higher in mice housed at thermoneutrality, although the difference reached statistical significance only in Cyp7b1^−/−^ mice (Figure 3C). Using gene expression analysis, we found that several fibrosis markers (including Timp1, Mmp2, Mmp12 and Mmp13) exhibited highest expression in the 30 °C-housed Cyp7b1^−/−^ mice (Figure 3D). 

Collectively, we observed that mice lacking CYP7B1 exhibit signs of increased MAFLD-related fibrosis and accumulate liver lipids associated with inflammation and insulin resistance [5,33] when housed at thermoneutral conditions.

### 3.3. CYP7B1 Deficiency Alters Hepatic Sterol Balance in MAFLD

**Figure 4:**
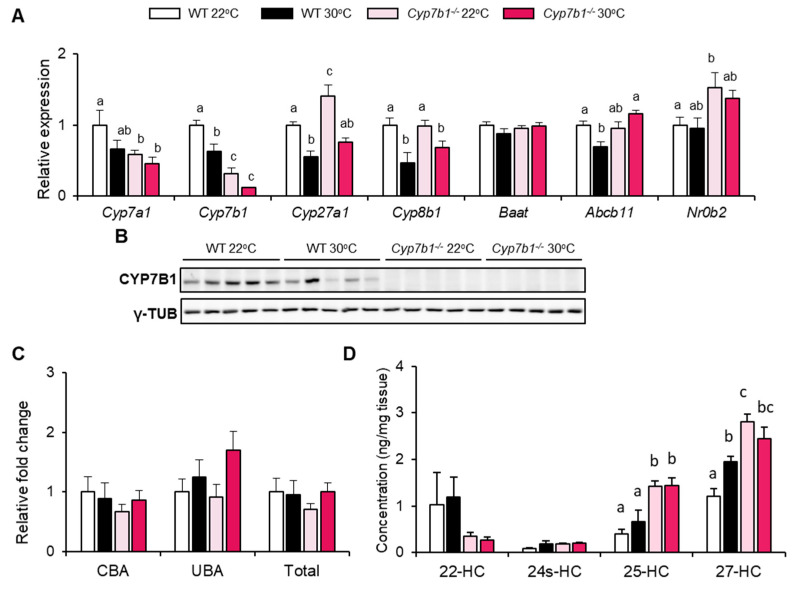
Influence of CYP7B1-deficiency on bile acid and oxysterol metabolism. Cyp7b1^−/−^ and WT littermates were housed at 22 °C or 30 °C and fed a CD-HFD for 8 months. (**A**) Relative hepatic expression of bile acid- and oxysterol-related genes (n = 8). (**B**) Western blot analysis of hepatic CYP7B1 and the loading control gamma tubulin (γ-TUB). (**C**) Relative change of portal bile acid levels in comparison to WT 22 °C mice (number of mice: WT 22 °C: n = 11; WT 30 °C: n = 9; Cyp7b1^−/−^ 22 °C: n = 16; Cyp7b1^−/−^ 30 °C: n = 13). (**D**) Levels of hepatic hydroxycholesterol species (n = 8). Data are shown as mean values ± SEM. Different letters indicate significant differences between groups (*p* < 0.05) determined by two-way ANOVA.

CYP7B1 is a critical enzyme in the alternative bile acid synthesis pathway and affects the biosynthesis of several hydroxycholesterols. Therefore, we investigated how its deficiency would affect hepatic expression of genes related to the metabolism of bile acids and hydroxycholesterols. The bile acid synthesis genes Cyp7a1, Cyp7b1, Cyp27a1 and Cyp8b1 were lower in WT mice housed at 30 °C compared to those housed at 22 °C (Figure 4A). In the Cyp7b1 knockout mice, where we detected a truncated Cyp7b1 mRNA at lower levels but no CYP7B1 protein (Figure 4B), a similar trend was observed for Cyp27a1 and Cyp8b1. In addition, at 22 °C Cyp7a1 exhibited lower expression in the Cyp7b1^−/−^ mice, which might be explained by an induction of its negative regulator Nr0b2 (Figure 4A). No major effects on gene expression were detected for bile acid-CoA:amino acid N-acyltransferase (BAAT) and ATP binding cassette subfamily B member 11 (ABCB11), proteins that mediate conjugation and biliary secretion of bile acids, respectively. 

Interestingly, CYP7B1 deficiency did not have a major impact on bile acid levels in portal blood (Figure 4C). In contrast to bile acids, a significant effect was observed for 27-HC and 25-HC (Figure 4D), the substrates of CYP7B1 that are generated by CYP27A1 and cholesterol 25-hydroxylase, respectively [20]. These sterols were higher in the livers of Cyp7b1^−/−^ mice while hydroxycholesterols that are not CYP7B1 substrates (22-HC, 24s-HC) were unaltered (Figure 4D). Overall, these data indicate that CYP7B1 primarily influences hydroxycholesterol levels but has little effect on bile acid metabolism in the dietary MAFLD model. 

### 3.4. Inflammatory Markers Are Increased in Cyp7b1^−/−^ Mice upon Thermoneutral Housing Conditions

**Figure 5:**
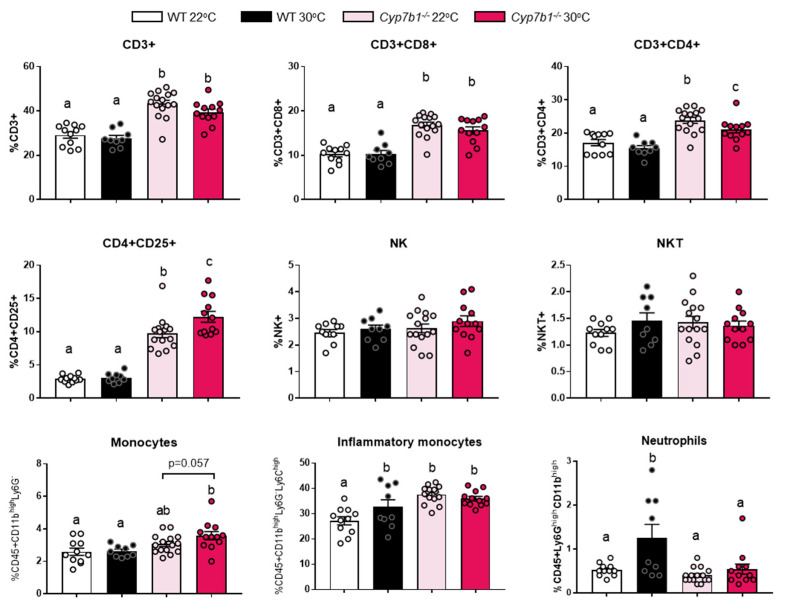
Immune cell profiling of the spleen. Flow cytometry was performed to determine CD3+, CD3+CD8+, CD3+CD4+ and CD4+CD25+ lymphocytes as well as NK, NKT, monocytes, inflammatory monocytes and neutrophils in Cyp7b1^−/−^ and wild littermates type housed under 22 °C or 30 °C, all fed a CD-HFD for 8 months. Data are shown as mean values ± SEM. (number of mice: WT 22 °C: n = 11; WT 30 °C: n = 9; Cyp7b1^−/−^ 22 °C: n = 15; Cyp7b1^−/−^ 30 °C: n = 12). Different letters indicate significant differences between groups (*p* < 0.05) determined by two-way ANOVA.

CYP7B1 deficiency affected hepatic lipid accumulation and fibrosis in thermoneutrality (Figure 2 and Figure 3). To investigate whether these pathological alterations were accompanied by specific inflammatory responses, we performed immune cell profiling via flow cytometry analysis of the liver and spleen. In the liver, we were unable to isolate a sufficient number of viable non-parenchymal liver, which probably can be explained by excess lipid deposition and fibrosis in our 8-month feeding study. In the spleen, flow cytometry analysis revealed that Cyp7b1^−/−^ mice had significantly increased numbers of total CD3+ T cells as well as the CD8+, CD4+ and CD4+CD25+ subpopulations (Figure 5). A similar trend was observed for Ly6G^−^ monocytes and Ly6G^high^ inflammatory monocytes but not for NK, NKT cells and neutrophils. Together, these results showed that CYP7B1-deficiency modulates immune cell subpopulations in the spleen, suggesting systemic effects on inflammation. 

**Figure 6:**
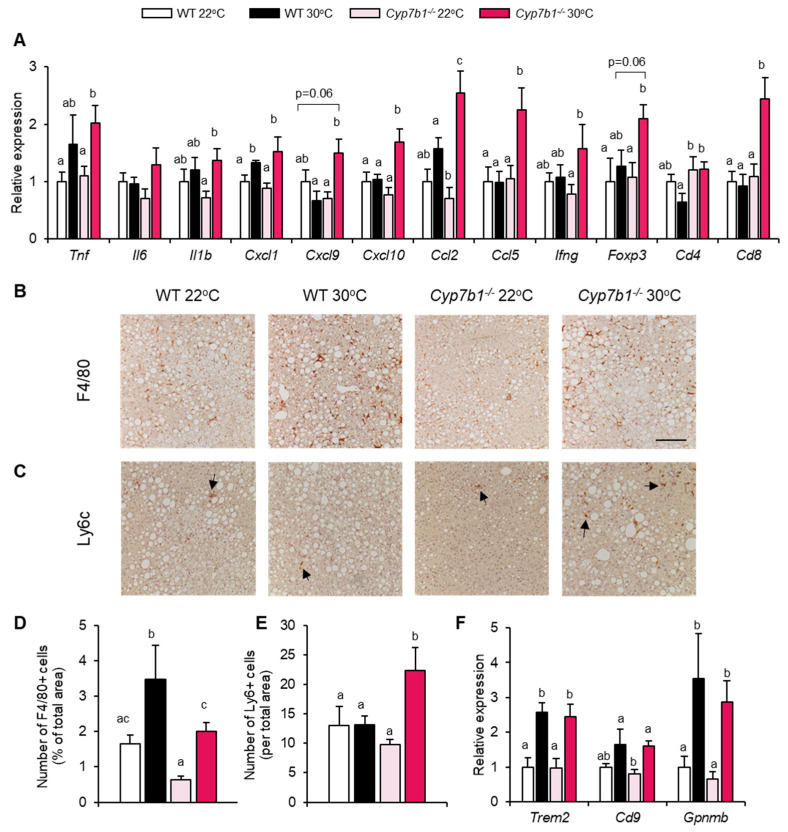
CYP7B1-deficiency leads to increased liver inflammation at thermoneutral housing conditions. Cyp7b1^−/−^ and WT littermates were housed at 22 °C or 30 °C and fed a CD-HFD for 8 months. (**A**) Relative hepatic expression of inflammation-related and T-cell related genes (n = 8). (**B**,**C**) Representative images of (**B**) F4/80+ cells and (**C**) Ly6c+ cells (scale bar 100 μm). (**D**,**E**) Quantifications of (**D**) F4/80+ and (**E**) Ly6c+ images shown in B and C, respectively (number of mice: WT 22 °C: n = 11; WT 30 °C: n = 9; Cyp7b1^−/−^ 22 °C: n = 16; Cyp7b1^−/−^ 30 °C: n = 13). (**F**) Relative hepatic gene expression of Kupffer cell-related markers (n = 8). Data are shown as mean values ± SEM. Different letters indicate significant differences between groups (*p* < 0.05) determined by two-way ANOVA.

To study the impact of CYP7B1 deficiency on immune cell infiltration into the liver, we performed hepatic immune cell profiling in Cyp7b1^−/−^ and WT mice housed at 22 °C or 30 °C after CD-HFD feeding for only three weeks. This time point represents an early stage of diet-induced MAFLD, enabling efficient organ disintegration and thus flow cytometry. As in spleen the most prominent differences were observed in T lymphocytes, we studied specifically hepatic T cell subpopulations (Figure S2). Characterization of liver-infiltrating T cells by flow cytometry did not show significant differences as depicted by the comparable CD3+, CD3+CD8+ and CD3+CD4+ subpopulations (Figure S2A). However, specific analysis of isolated T cells re-stimulated with PMA/ionomycin showed that IL17- and IFN-producing CD4+ cells from Cyp7b1^−/−^ mice trended to be higher. Moreover, at thermoneutrality, TNF-producing CD4+ cells were significantly elevated in CYP7B1-deficient mice in comparison to their WT littermates (Figure S2B). The respective CD8+ cytokine producing cells were unaltered (Figure S2C). These data indicate that short-term CD-HFD feeding already causes subtle changes in T cell sub-populations in livers of CYP7B1-deficient mice. To study whether CYP7B1 deficiency has effects on liver inflammation in the chronically fed mice, we analyzed the hepatic expression of inflammation-related genes including cytokines, chemokines and lymphocyte markers in WT and Cyp7b1^−/−^ mice housed either at 22 °C or 30 °C. Most of the genes investigated exhibited higher expression in mice housed at 30 °C compared to those housed at 22 °C, an effect that was more pronounced in the Cyp7b1^−/−^ mice than in the WT mice for Cxcl10, Ccl2, Ccl5, Cd4 and Cd8 (Figure 6A). Consistent with elevated Cd4 and Cd8, a higher number of CD3-positive cells was detected via immunohistochemistry (Figure S3). As CCL2 is a critical monocyte chemoattractant, we determined infiltration of proinflammatory monocytes by immunohistology. In comparison to tissue-resident F4/80-positive Kupffer cells (Figure 6B,D), we found increased numbers of infiltrating Ly6c-positive monocytes specifically in livers of Cyp7b1^−/−^ mice housed at thermoneutrality (Figure 6C,E). A previous study showed an expansion of specific Kupffer cells characterized by high expression of Trem2, Cd9, and Gpnmb in NASH livers [34]. In line with this study and also with the F4/80 staining (Figure 6D), we detected higher expression of these genes at thermoneutrality (Figure 6F).

Altogether, these results indicate that proinflammatory immune responses are exaggerated in livers of mice lacking CYP7B1 that are housed at thermoneutrality. Moreover, CYP7B1 deficiency primarily influences immune cell infiltration but not expansion of tissue-resident Kupffer cells. 

### 3.5. Compensatory Lipoprotein Receptor Upregulation in CYP7B1-Deficient Mice at Room Temperature

**Figure 7:**
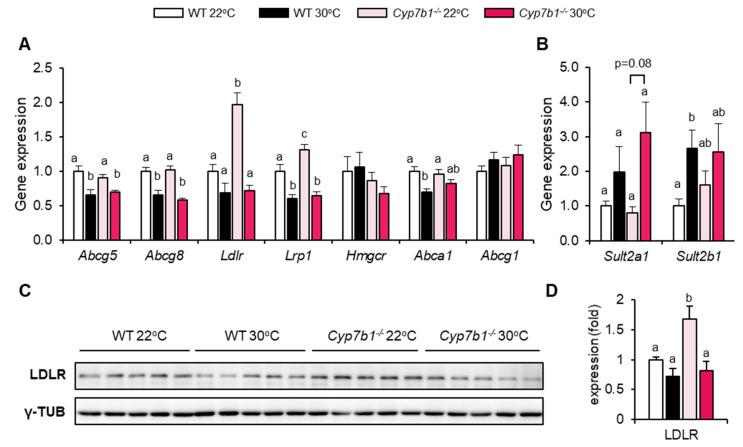
CYP7B1-deficiency affects expression of lipoprotein receptors but not other cholesterol-handling genes. Cyp7b1^−/−^ and WT littermates were housed at 22 °C or 30 °C and fed a CD-HFD for 8 months. (**A**,**B**) Relative hepatic expression of (**A**) lipoprotein receptors and cholesterol metabolism-related genes and (B) hepatic sulfotransferases (n = 8). (**C**) Western blot analysis and (**D**) quantification of hepatic LDLR. Gamma tubulin (γ-TUB) was used as a loading control and for normalization. Data are shown as mean values ± SEM. Different letters indicate significant differences between groups (*p* < 0.05) determined by two-way ANOVA.

We observed that CYP7B1-deficiency leads to lipid accumulation, proinflammatory and profibrotic responses only under thermoneutral housing conditions. Given the known role of cholesterol for MAFLD development, we studied expression of cholesterol handling proteins (Figure 7). The liver X receptor (LXR) positively regulates the transcription of hepatic cholesterol export pumps including dimeric ABCG5/ABCG8, ABCA1 and ABCG1 [35]. Despite the elevation of the LXR agonists 25-HC and 27-HC (Figure 4), expression of these genes was not affected by CYP7B1 deficiency (Figure 7A), arguing against activation of LXR under this condition. This may be explained by increased production of sulfated hydroxysterols that can act as LXR antagonists [36], as suggested by a trend for higher expression of the oxysterol sulfotransferases Sult2a1 and Sult2b1 at thermoneutrality (Figure 7B).

Notably, Cyp7b1^−/−^ mice housed at 22 °C but not at 30 °C exhibited higher liver expression of the LDL receptor (LDLR) both at the mRNA (Figure 7A) and the protein (Figure 7C,D) level. Similarly, the LDLR-related protein-1 (Lrp1) was expressed at higher levels in these mice. No regulation was observed for hydroxyl-methylglutaryl-CoA reductase (Hmgcr), suggesting that sterol-regulatory element-binding protein-2 that positively regulates both Ldlr and Hmgcr is not responsible for the LDLR upregulation in Cyp7b1^−/−^ mice housed at 22 °C. 

Taken together and in line with experiments performed in LDLR deficient mice [37], these data suggest that increased flux of lipoprotein-associated lipids into hepatocytes is protective in the context of MAFDL at 22 °C. On the other hand, hepatic lipoprotein receptor expression is not induced in CYP7B1-deficient mice at thermoneutrality, favoring proinflammatory and fibrotic responses, possibly due to lipid and oxysterol accumulation in non-parenchymal and immune cells. 

## 4. Discussion

Changes in bile acid and oxysterol levels contribute and/or occur during the establishment of metabolic abnormalities such as type 2 diabetes and MAFLD [19,38]. Therefore, unravelling the contribution of specific cytochrome P450 superfamily enzymes that mediate their biosynthetic pathways can shed light on their pathophysiological role. Interestingly, the alternative bile acid synthesis pathway seems to be differentially altered in metabolic-related disorders, since CYP7B1 has been described as decreased in murine models as well as patients with type 2 diabetes [10,39,40] but was found elevated in patients with progressive MAFLD [41]. Of note, the results of mechanistic studies investigating the role of CYP7B1 in MAFLD are quite inconsistent. In a recent study, Raselli et al. pointed towards a non-essential role of CYP7B1 in MAFLD progression, as both hepatic fibrosis and disease activity score were comparable between HFD-fed WT and Cyp7b1^−/−^ mice [42]. On the contrary, Kakiyama et al. provided evidence that, especially under conditions of insulin resistance, CYP7B1 could mediate progression to steatohepatitis, owing to the accumulation of toxic oxysterols that could promote inflammation and liver damage [43]. Recently, it has been shown that thermoneutral housing, a condition of low brown adipose tissue activity and diminished energy expenditure [44], provides a suitable model of exacerbated diet-induced MAFLD in mice [23]. Both Raselli et al. and Kakiyama et al. studied mice housed only at conventional room temperature. As CYP7B1 is regulated by ambient temperature [10], we aimed to understand the role of the alternative bile acid pathway in MAFLD at different housing temperatures. In the current study, we found that hepatic Cyp7b1 expression is significantly higher in WT mice housed at 22 °C compared to 30 °C. This indicates that the alternative route of BA synthesis is higher at lower ambient temperatures, which is associated with enhanced hepatic cholesterol metabolism and accelerated lipoprotein clearance [8,9,30]. Together, these processes could explain the milder MAFLD phenotype of conventionally housed mice when compared to mice housed at thermoneutrality [23]. In line with Raselli et al. [42], CYP7B1 deficient mice housed at 22 °C have a disease phenotype upon CD-HFD feeding largely indistinguishable from their WT littermates housed at the same temperature. In contrast, we showed that Cyp7b1^−/−^ mice housed at 30 °C are characterized by exacerbated MAFLD. These data indicate that CYP7B1 activity counteracts disease progression specifically under conditions of low thermogenic stress, representing typical human living conditions [20,22]. Future studies measuring parameters such as core body temperature, energy expenditure and heat loss would find it important to delineate how CYP7B1-dependent hydroxycholesterols and/or bile acids influences diet-induced energy homeostasis in the context of NASH progression. 

The alternative pathway is described as a minor contributor in the biosynthesis of bile acid compared to the classical one. Previously, we have shown that hepatic CYP7B1 mRNA was moderately reduced in human liver samples of obese diabetic compared to non-obese subjects [10]. Recent publications aiming at the identification of hepatic transcriptomic signatures distinguishing simple steatosis from NASH did not report differential mRNA expression of CYP7B1 when comparing these groups [45,46]. However, the expression data do not exclude the possibility that alterations in CYP7B1 activity may aggravate MAFLD in humans. Supporting this possibility, newborns with loss of function CYP7B1 mutations develop severe liver phenotypes [47,48]. These patients lacking CYP7B1 activity are characterized by elevated levels of 24-HC, 25-HC, 27-HC, 3β-hydroxy-5-cholenoic and 3β-hydroxy-5-cholestenoic acids, aggressive hepatic fibrosis and inflammation that eventually lead to liver failure and premature death. Similarly, in our study we show that CYP7B1 deficiency in rodents leads to hepatic accumulation of the enzyme’s specific hydroxycholesterol substrates 25-HC and 27-HC. All in all, the 30 °C-housed Cyp7b1^−/−^ mice seem to present a hepatic milieu characterized by the accumulation of specific hydroxycholesterols. On the other hand, our data show that the 22 °C-housed Cyp7b1^−/−^ mice display upregulation of Cyp27a1 that may explain the slightly higher levels of its respective hydroxycholesterol product 27-HC. Interestingly, Bieghs et al. described that administration of 27-HC in high-fat- high cholesterol diet-fed mice can lower cholesterol-induced inflammation and attenuate steatohepatitis over time [49]. This feature of 27-HC could, at least in part, contribute to the milder MAFLD-related phenotype in the conventional housed Cyp7b1^−/−^ mice. Another explanation could be the upregulation of both Ldlr and Lrp1 in CYP7B1 deficient mice housed at 22 °C but not at thermoneutrality. These lipoprotein receptors have been shown to protect against diet-induced MAFLD progression in mice [32,46,47]. Accordingly, low expression of Ldlr and Lrp1 at thermoneutrality may favor lipotoxic responses mediated by accumulating lipoproteins and oxysterols. 

Lipid species such as oxysterols have potent immunomodulatory functions, as they can activate several nuclear receptors (such as liver X receptors (LXRs) and retinoic acid orphan receptors (RORs)). Next to their role in regulating cholesterol and energy metabolism, these transcription factors are known to influence immune functions (including macrophage phagocytosis, T- and B-lymphocyte activation, immune cell migration and polarization [50]. Cell types of both the innate and the adaptive immune systems are important mediators of MAFLD progression [51,52]. Here, we observed a trend for increased numbers of hepatic IL17-, TNF- and INF-producing CD4+ lymphocytes in CYP7B1 deficient mice in the early stages of MAFLD progression under thermoneutral conditions. In later stages, liver stiffness and hepatic lipid accumulation prevented immune cell profiling via flow cytometry. However, immunohistology and expression analysis of inflammatory genes provided evidence that the release of specific cytokines and chemokines such as CCL2 and CCL5 by liver cells promoted the recruitment of T cells and inflammatory LyC6+ monocytes in thermoneutral housed CYP7B1-deficient mice. Importantly, these chemokines have been described to be elevated in MAFLD patients and to be important for the progression MAFLD in murine models [53,54]. Future studies are warranted to delineate the detailed mechanisms and the relevance of specific oxysterols for the regulation of immune cell subtypes in MAFLD. 

In conclusion, our study demonstrates the relevance of CYP7B1 for the prevention of MAFLD and associated immunometabolic disturbances in the context of thermoneutrality. Moreover, our data suggest that stimulation of hepatic LDL receptor activity by approved pharmacological agents such as statins or PCSK9 antagonists are a testable strategy for the treatment of MAFLD in humans. 

## Data Availability

The data presented in this study are openly available in Mendeley (https://data.mendeley.com, accessed on 4 October 2021) at DOI:10.17632/5xkrbjbb9m.1.

## Supplementary Materials

The following items are available online at https://www.mdpi.com/article/10.3390/cells10102656/s1, Figures S1 (Relative expression of thermogenesis-related genes in brown adipose tissue) and S2 (CYP7B1-deficiency affects hepatic cytokine-producing T cell populations during the onset of MAFLD in thermoneutral conditions).
