# Supplementary material for: Oxysterol 7-α Hydroxylase (CYP7B1) Attenuates Metabolic-Associated Fatty Liver Disease in Mice at Thermoneutrality
Source: Cells. 2021 Oct 5;10(10):2656. doi: 10.3390/cells10102656 (PMC8534379; doi:10.3390/cells10102656)
Supplement: Supplementary file 1 [file cells-10-02656-s001.zip › cells-1319972-supplementary.pdf]

Figure S1

A.

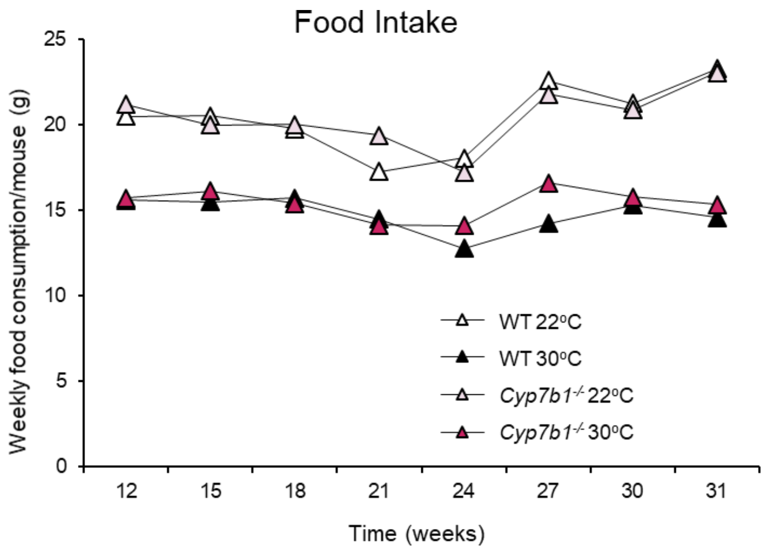

B.

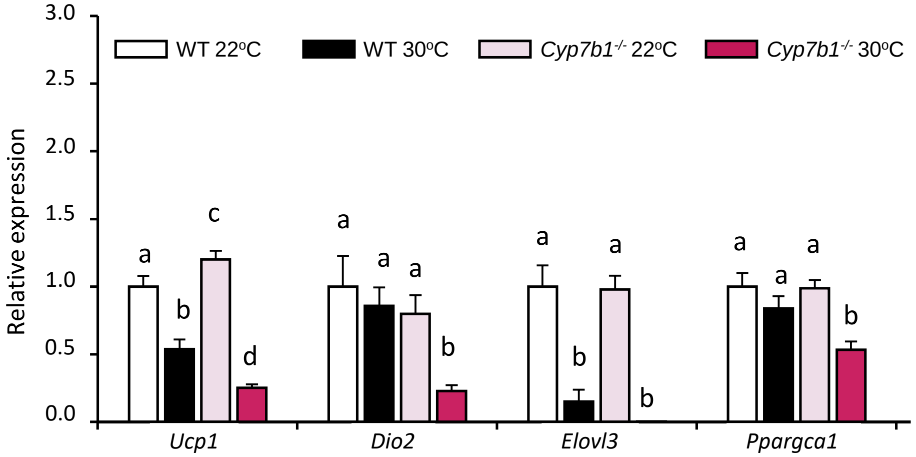

Figure S1. (A) *Cyp7b1*$^{-/-}$ and WT littermates housed under 22 °C or 30 °C were fed a CD-HFD for 8 months. (A) Food intake (WT 22 °C: n= 11; WT 30 °C: n=9; *Cyp7b1*$^{-/-}$ 22 °C: n=16; *Cyp7b1*$^{-/-}$ 30 °C: n=13). (B) Relative expression of thermogenesis-related genes in brown adipose tissue. Data are shown as mean values ± SEM. n=8 per group, different letters indicate significant differences between groups ($p<0.05$) determined by two-way ANOVA.

Figure S2

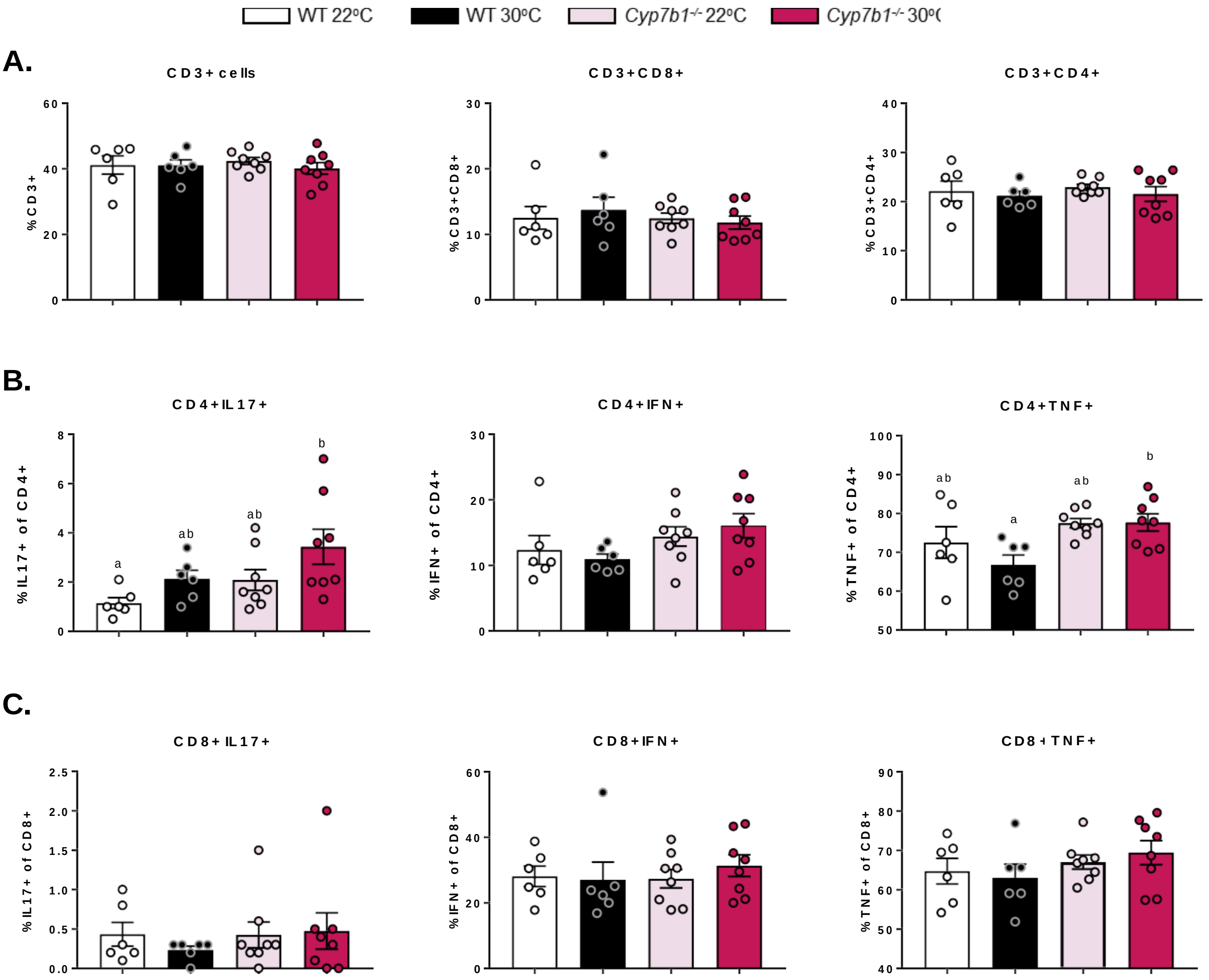

Figure S2. CYP7B1-deficiency affects hepatic cytokine-producing T cell populations during the onset of MAFLD in thermoneutral conditions. FACS analysis of (A) all CD3+ lymphocytes, as well as IL17-, IFN-, TNF-producing (B) CD3+CD8+ and (C) CD3+CD4+ cells in livers of *Cyp7b1*$^{-/-}$ and wild type littermates housed under 22ºC or 30ºC, all fed a CD-HFD for 3 weeks. Data are shown as mean values ± SEM. n=6-8/group. Different letters indicate significant differences between groups ($p<0.05$) determined by two-way ANOVA.

Figure S3

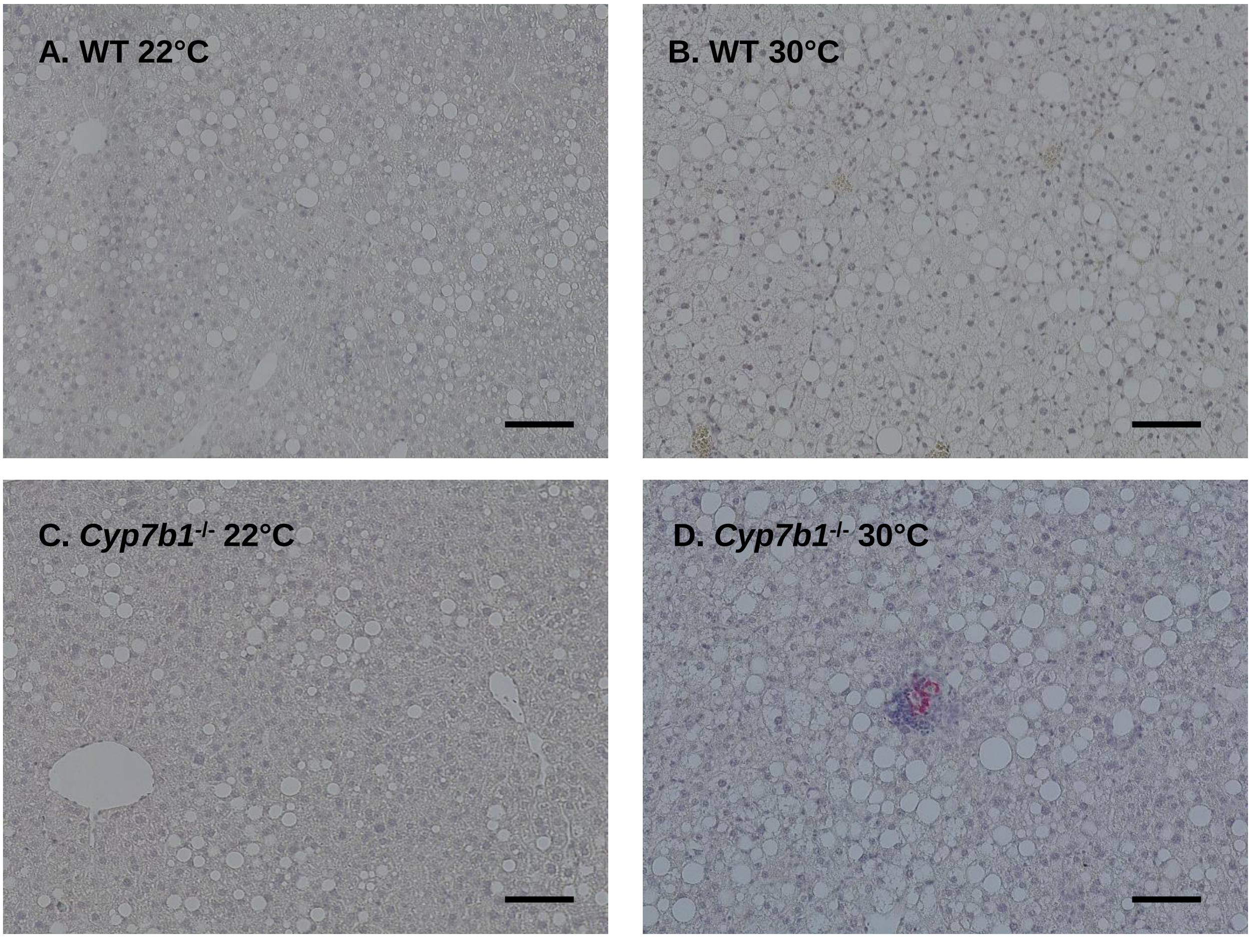

Figure S3. Immune cell infiltration in CYP7B1-deficient mice housed at thermoneutrality. *Cyp7b1*$^{-/-}$ and WT littermates housed under 22 ℃ or 30 ℃ were fed a CD-HFD for 8 months. Representative images of CD3 immunostaining in livers of (A) WT 22 ℃, (B) WT 30 ℃, (C) *Cyp7b1*$^{-/-}$ 22 ℃, and (D) *Cyp7b1*$^{-/-}$ 30 ℃. In two out of eight liver sections from WT 22 ℃, in two out of eight liver sections from WT 30 ℃, one out of eight liver sections from *Cyp7b1*$^{-/-}$ 22 ℃, and four out of eight liver sections from *Cyp7b1*$^{-/-}$ 30 ℃ CD3 could be detected by immunohistochemistry. Bar 100 μm.
